# Confronting medical grifting: Fraudulent and unproven products and interventions in apheresis, transfusion and biotherapies

**DOI:** 10.1111/bjh.70194

**Published:** 2025-10-11

**Authors:** Brian D. Adkins, Sheharyar Raza, Victoria Costa, Elizabeth S. Allen, Laura D. Stephens, Jennifer S. Woo, Garrett S. Booth, Jeremy W. Jacobs

**Affiliations:** ^1^ Department of Pathology University of Texas Southwestern Medical Center Dallas Texas USA; ^2^ Department of Laboratory Medicine and Pathobiology University of Toronto Toronto Ontario Canada; ^3^ Medical Affairs and Innovation Canadian Blood Services Toronto Ontario Canada; ^4^ Department of Pathology NYU Grossman School of Medicine New York New York USA; ^5^ Department of Pathology University of California San Diego La Jolla California USA; ^6^ Department of Pathology City of Hope National Medical Center Irvine California USA; ^7^ Department of Pathology, Microbiology, and Immunology Vanderbilt University Nashville Tennessee USA

**Keywords:** apheresis, biotherapy, ethics, misinformation, transfusion medicine

## Abstract

Medical scams or grifting has long been a societal issue, though in recent years, the problem has become increasingly mainstream, especially as it relates to transfusion medicine, apheresis and biotherapies. Unfortunately, unsubstantiated medical claims, or unfounded interventions such as therapeutic plasma exchange for longevity or dangerous stem cell injections, are leading to significant medical waste and patient harm. Herein, we review contemporary medical grifts, cognitive biases and present a framework for managing these issues to ensure legitimate care and safety for patients.

## Graphical abstract

A basic overview of medical grifting for haematologists with practical guidance in identification and tips for management.

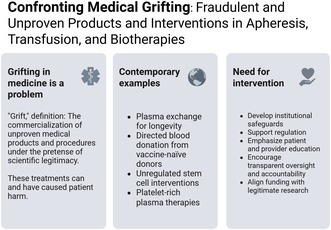

## INTRODUCTION

Public trust in medical professionals is foundational to effective healthcare delivery. Yet in recent years, this trust has been steadily undermined by the spread of misinformation, fuelled by social media and deliberate marketing.^1^, ^2^ The resulting erosion of confidence in traditional medical systems has created fertile ground for exploitation by individuals and companies promoting unproven or fraudulent health interventions—often referred to colloquially as ‘grifters’.^3^ While the term ‘grift’ can refer to small‐scale swindling, we define it as the commercialization of unproven medical products and procedures under the pretence of scientific legitimacy. These interventions are typically marketed as innovative or evidence‐based but lack rigorous clinical validation and are frequently driven by financial incentives rather than therapeutic benefit. The transfusion medicine, apheresis and biotherapy fields have not been immune to these developments. Notable examples include the use of therapeutic plasma exchange (TPE) for anti‐ageing, off‐label platelet‐rich plasma (PRP) injections, solicitation of blood donations from vaccine‐naïve individuals and unlicensed stem cell infusions marketed for regenerative purposes.

Although often perceived as fringe, these interventions are increasingly prevalent—and, in some cases, tacitly endorsed by influential figures in medicine, media and politics. In the absence of a coordinated response from clinicians, health systems and professional societies, this trend threatens to further reduce public trust, jeopardize patient safety and blur the distinction between science and pseudoscience. This review explores the rise of biologics‐related health fraud within the domains of transfusion medicine, apheresis and biotherapies. In this review, we highlight representative examples and examine the financial incentives and psychological mechanisms that sustain these practices. We also analyse the regulatory gaps and sociopolitical forces that enable their proliferation and discuss the ethical challenges they pose for providers and institutions. Finally, we propose strategies for distinguishing legitimate medical innovation from opportunistic exploitation and offer practical guidance for confronting unfalsifiable claims that undermine the foundations of evidence‐based care.

## HISTORICAL AND CONTEMPORARY MEDICAL GRIFTING

Medical grifting is not a new phenomenon—it has merely evolved alongside technology, regulation and public fears. Although certain historic interventions such as blood‐letting for bad humours or transfusion of milk might appear fraudulent today, some of these bizarre practices were based on well‐intended contemporary medical theory; however, using medical interventions for monetary gain is an old practice.^4^ In the 19th century, travelling medicine shows peddled ‘cure‐alls’ infused with alcohol, narcotics or radioactive substances, often marketed with exotic ingredients and pseudo‐medical claims.^5^, ^6^, ^7^, ^8^ As mass media expanded, patent medicines and ‘snake oil’ remedies advertised to ‘cleanse the blood’ or ‘cure cancer’ found new audiences through print and radio.^6^ Even as landmark regulations such as the Pure Food and Drug Act or the Public Health Service Act as well as the creation of the US Food and Drug Administration (FDA) sought to curb these abuses, grifters have always adapted—shifting tactics to stay one step ahead.^9^

Contemporary iterations of medical grift are seemingly more polished. With direct‐to‐consumer shopping evolving in the late 20th century and the permissive marketplace created by the Dietary Supplement Health and Education Act of 1994, which allows for supplements to be distributed without FDA oversight, avenues for unproven interventions continue to remain open.^10^ Today, unproven biologics and therapies are marketed under the guise of biotechnology, regenerative medicine or precision health. Social media has amplified these messages.^11^ Patients with serious illnesses, including cancer, increasingly report delaying evidence‐based care in favour of ‘natural’ remedies, such as restrictive diets, essential oils or high‐dose vitamin infusions promoted by online influencers—sometimes with devastating outcomes.^12^, ^13^ In the realm of anti‐ageing and regenerative therapies, private clinics may charge from $16,500 to $25,000 per dose for unregulated stem cell injections, often sourced from low‐wage donors.^14^ In the United Kingdom, cosmetic clinics have marketed exosome facials containing human‐derived biologics—banned due to safety concerns—with promises of skin rejuvenation and hair regrowth.^15^, ^16^ In the United States, lay‐press articles have recently covered claims that TPE promotes longevity, with purveyors claiming it is equivalent to changing the oil in a car.^17^, ^18^

These modern grifts exploit the language and aesthetics of legitimate science, appropriating terms like ‘cellular therapy’, ‘precision medicine’ and ‘personalized treatment’ to lend an air of credibility. This pattern reflects a historical continuum of medical exploitation—today's biologics‐based scams are not novel; instead, they simply represent the latest iteration in a long‐standing tradition of health fraud. The addition of scientific buzzwords to otherwise poor explanations makes them seem more satisfying to laypeople—even when they diminish understanding—because individuals unconsciously infer that the jargon bridges explanatory gaps.^19^

## IMPLICATIONS AND HARMS

The risks associated with unproven biologics vary by product and procedure, encompassing both direct and indirect harms, as well as significant ethical, economic and societal implications (Table 1).

**TABLE 1:** Representative unproven biologics‐based interventions in transfusion medicine, apheresis and cellular therapy.

Intervention	Claimed indications (not exhaustive)	Marketing rationale	Known or potential harms
Therapeutic plasma exchange for ageing/longevity	Longevity, youth restoration	Framed as ‘changing your blood oil’, linked to biomarker normalization	Hypocalcaemia, iron‐deficiency anaemia, allergic reactions, hypotension, infection, coagulation factor and immunoglobulin depletion, financial cost and potential for resource diversion^20^, ^21^, ^22^, ^23^, ^24^, ^25^, ^26^, ^27^, ^28^
Vaccine‐naïve blood transfusion	‘Uncontaminated’ or ‘pure’ blood	Distrust of mRNA vaccines, anecdotal safety concerns	Resource diversion, increased infectious disease risk from first‐time donors, unused units, impacts on the community blood supply, financial cost^29^, ^30^, ^31^
Off‐label platelet‐rich plasma (PRP)	Neurological recovery, anti‐ageing, arthropathies, tendinopathies, cosmetics, fertility	Promoted as ‘natural healing’, bolstered by celebrity endorsements	Local infection, financial exploitation, delay of effective treatment^32^, ^33^
Stem cell infusions (unlicensed)	ALS, spinal cord injury, erectile dysfunction	‘Regeneration’, ‘cellular repair’, stem cell tourism	Financial exploitation, tumorigenesis, immune reactions, infection, spinal masses, blindness, paralysis, death^34^, ^35^, ^36^, ^37^
Exosome injections for hair loss	Hair regrowth, scalp rejuvenation	Buzzword‐heavy marketing using ‘nanoparticles’, ‘biologic communication’	Financial exploitation, unknown systemic effects, sterility concerns, lack of regulation, substantial financial cost^15^, ^16^

### Therapeutic plasma exchange for longevity

In the case of TPE marketed for life extension, no controlled human studies substantiate its efficacy. Existing data are limited to biomarker shifts consistent with nonspecific plasma protein removal. Whether such changes confer clinical benefit remains unproven.

While TPE is generally well tolerated for established indications, it carries procedural risks—including hypocalcaemia, volume shifts, vascular access complications and infection.^20^, ^21^, ^22^, ^23^, ^24^ Repeated treatments may also result in cellular loss in the apheresis circuit and iron deficiency anaemia, as well as potential immunodeficiency or coagulopathy from the nonselective depletion of immunoglobulins and clotting factors.^25^, ^26^, ^27^, ^28^ Beyond physical harm, promoting TPE for anti‐ageing purposes risks distorting public understanding of its legitimate, life‐saving use in conditions like thrombotic thrombocytopenic purpura or myasthenia gravis exacerbations.^38^, ^39^, ^40^ In many global settings, TPE remains a scarce resource, and its misuse may divert plasma products or albumin away from patients with evidence‐based needs.

The American Society for Apheresis (ASFA) *guidelines* assign each indication a Category (I–IV) for clinical appropriateness and a Grade (1A–2C) for strength/quality of evidence (I = first‐line; II = second‐line/adjunct; III = role not established; IV = ineffective/harmful). ‘Longevity’/’anti‐ageing’ is not an ASFA‐recognized indication.^40^ When such requests arise, clinicians should confirm whether the underlying condition appears on an ASFA fact sheet and documents its Category/Grade if applicable; explain that anti‐ageing TPE lacks an assigned indication and outcomes evidence; and channel non‐indicated use through institutional apheresis/ethics review or IRB‐approved research. Using this framework helps distinguish legitimate, evidence‐based apheresis from speculative, direct‐to‐consumer applications.

### Directed donation from vaccine‐naïve donors

Requests for blood donations from unvaccinated individuals present another example of potential harm.^29^, ^41^, ^42^ These requests consume substantial healthcare resources: staff spend days navigating logistics, fielding concerns and coordinating with blood suppliers, without clinical justification. Directed donations also tend to come from first‐time donors, who are more likely to test positive for transfusion‐transmissible infections.^30^, ^31^ For this reason, many hospitals decline to release unused directed donor units into the general inventory, leading to waste, with one study reporting nearly a 70% discard rate among directed donations for paediatric surgery.^43^ These efforts frequently culminate in the collection of blood that is often discarded, redirecting staff time and institutional resources away from evidence‐based patient care.

Meanwhile, organizations such as *Safe Blood* or *Blessed By His Blood* create membership subscription models and sell merchandise to co‐ordinate efforts under the guise of patient autonomy, turning ideology into revenue.^44^ *Safe Blood*, for example, has a subscription service with a $40 sign‐up fee and $10 monthly rates to receive unvaccinated blood; it also offers a one‐time payment option of $1000, while personal information is shared or sold to third parties for advertising, requiring subscribers to opt out to prevent spam calls or e‐mail.^45^

### Unregulated stem cell interventions

Stem cell infusions for unapproved conditions including Alzheimer's disease, muscular dystrophy, autism, spinal cord injury and even COVID‐19 have caused significant harm—ranging from paralysis to infections, blindness and death.^34^, ^35^, ^36^, ^46^ A notable case in *The New England Journal of Medicine* described a spinal cord tumour following international ‘stem cell tourism’.^37^ These complications are often secondary to inadequate and unregulated cell processing techniques, improper storage conditions and unsafe administration practices.^36^ While many of these interventions are performed with mesenchymal stem cell products, exposure to foreign cells additionally places patients at risk for human leukocyte antigen (HLA) sensitization; if haematopoietic stem cells are used, there is a theoretical risk for red cell antigen sensitization or blood typing issues moving forward.

### Platelet‐rich plasma therapies

In addition to orthopaedic applications, PRP injections are widely promoted for a range of other indications like hair restoration and cosmetic procedures, despite limited high‐quality controlled trials demonstrating durable clinical benefit.^32^, ^47^, ^48^ Most PRP protocols lack standardization in platelet concentration and delivery techniques, leading to inconsistent outcomes and hampering reproducibility.^47^, ^49^, ^50^ Procedural risks include pain, bruising and haematoma at injection sites, as well as limb swelling or tissue necrosis if administered improperly.^33^ Furthermore, PRP treatments often occur in nonmedical settings or by practitioners without formal training in aseptic technique, raising the risk of local infections and, in rare instances, blood‐borne pathogen transmission. A Centers for Disease Control and Prevention (CDC) investigation into ‘vampire facial’ procedures with PRP injections at a medical spa demonstrated inadequate aseptic technique, inadequate cleaning, unlabelled specimens and mishandling of sharps, which led to unnecessary HIV exposure, resulting in a cluster of HIV transmission occurring in five individuals.^32^

Notably, Medicare does provide national coverage for autologous PRP to treat chronic, non‐healing diabetic wounds for up to 20 weeks when prepared with FDA‐cleared devices indicated for exuding cutaneous wounds.^51^ After 20 weeks—and for other chronic, non‐healing wounds—coverage is not set nationally; it is determined by the beneficiary's Medicare Administrative Contractor (MAC), via a Local Coverage Determination or case‐by‐case review, and therefore varies by jurisdiction. By contrast, PRP for musculoskeletal and joint conditions, and for cosmetic purposes is generally non‐covered; therefore, *in the absence of supportive evidence*, wound‐care coverage should not be generalized to orthopaedic or aesthetic applications.

### Economic and societal costs

Beyond individual harm, medical grifting imposes systemic economic burdens. Patients often pay out of pocket—sometimes tens of thousands of dollars—for unproven treatments marketed by concierge clinics and alternative wellness centres. When these interventions fail or cause complications, the formal healthcare system absorbs downstream costs, including diagnostic workups and medical management. Using medical spas as an example, while the precise costs of these downstream effects remain a gap in the literature, debilitating adverse effects from improper injection or contaminated injections could lead to serious patient harm including local injury or transmission of infectious disease requiring long‐term management from traditional medical practices; the regulatory landscape is scattered at best and may result in out‐of‐pocket payment or Medicare/Medicaid paying for these adverse outcomes.^52^

When clinicians or institutions acquiesce to grift‐related requests—due to lack of policy clarity or institutional pressure—it may inadvertently confer legitimacy. Such actions strain staff, lead to inefficient use of institutional resources, erode public trust, confuse the public about the role of cellular therapies and may hinder clinical trial enrolment or donor recruitment, further undermining the healthcare and biomedical research systems. Institutional credibility, once lent to fraudulent care, is difficult to reclaim.

### Ethical and legal considerations

Grift‐related demands place clinicians in ethically precarious positions. Appeals are often framed as exercises of patient autonomy, but honouring unproven requests may violate the ethical duty to avoid harm. Balancing autonomy against beneficence and non‐maleficence is particularly difficult when the intervention carries risk and no demonstrable benefit. At the same time, denying such requests can damage therapeutic relationships, especially when patients interpret refusal as ideological or paternalistic. Furthermore, as licensed healthcare professionals, physicians owe a legal and ethical fiduciary duty to place each patient's best interest above all other considerations—including any consulting relationships—and must advise against pursuing interventions for which there is insufficient evidence, even if they serve in a role that stands to benefit from pursuing such an intervention.

Providers must also navigate institutional expectations, reputational concerns and potential legal implications. The cumulative effect is a professional environment where clinicians are expected to counter misinformation, protect finite resources and maintain trust—all without consistent policy support or institutional protection.

## WHY THESE INTERVENTIONS PERSIST

### Profit, belief and hope

The growth of biologics‐driven grift is fuelled by a potent blend of financial opportunism and genuine belief. While many purveyors of unproven therapies are driven by profit, others may earnestly believe in the efficacy of their interventions despite lacking rigorous evidence. This convergence fosters a self‐sustaining system: clinics charge thousands to tens of thousands of dollars for therapies marketed with sophisticated language, while patients, desperate for relief, often pay out‐of‐pocket. Entrepreneurs have exploited weak or vague regulatory oversight to build billion dollar industries—concierge wellness centres, free‐standing apheresis clinics, regenerative medicine boutiques and direct‐to‐consumer platforms that operate in legal grey zones and target vulnerable populations.^53^, ^54^, ^55^

### Marketing tactics and cognitive traps

Grifters deploy polished marketing strategies designed to mimic legitimacy. These include scientific sounding terminology (e.g. ‘cellular optimization’, ‘exosomes’), the bundling of unproven therapies with common sense health advice such as improved diet and exercise, emotionally charged patient testimonials and celebrity endorsements. Social media platforms act as amplifiers, reinforcing narratives through repetition and selective anecdote.

These tactics are especially effective because grifters often exploit well‐known cognitive biases that underlie patient decision‐making. Confirmation bias, whereby people give undue weight to new information that confirms a pre‐existing belief, is often used to build on pre‐existing scepticism about mainstream medicine.^56^, ^57^ For instance, businesses claim that plasma exchange uses the body's ‘natural’ repair mechanisms to reverse ageing.^58^ A different tactic is to leverage authority bias, whereby endorsements—like claims from an influential health figure about their own stem cell treatment—lend credibility in lieu of hard scientific evidence.^59^, ^60^ Additionally, emotionally charged success stories create potent memories that are susceptible to the availability heuristic, wherein individuals rely on the information that most readily comes to mind. For example, a gold medal performance after stem cell injections for an athletic injury may be used to promote unsubstantiated therapies to injured athletes.^61^ These and other biases are powerful because they can influence medical decision‐making merely by reframing the way facts are presented, often without having to rely on explicit disinformation or directly refute peer‐reviewed research.^62^

Compounding these biases, humans are often worse at recalling *where* they learned a fact even when they vividly remember the fact itself.^63^ According to the source monitoring framework, people frequently retain content but lose track of its origin, so statements from unreliable purveyors can persist unchallenged. Repeated exposure—especially via social media feeds—further erodes source attributions, reinforcing misremembered claims and making corrective interventions far less effective.^64^ Repetition across platforms thus acts like an ‘origin eraser’, letting misinformation circulate untethered from its unreliable source and resurface later as an apparently free‐standing fact, further reinforcing the grifter's narrative.

Furthermore, many of the sellers of these unproven therapies appeal to desperation, targeting their products to individuals with chronic or terminal illness who may be particularly vulnerable, especially when conventional medicine offers limited options. Finally, by framing benefits as subjective (e.g. ‘you'll feel younger’), purveyors make it practically impossible to disprove their assertions. Together, these strategies create a persuasive illusion of legitimacy, making grifting difficult to detect—and harder to challenge.

### Appeal to ‘natural’ self‐healing

Unproven interventions appear to be disproportionately concentrated in biotherapies, potentially because they tap a powerful *naturalness bias*: the intuitive belief that substances drawn from one's own body (or from plants) are inherently safer and more wholesome than ‘foreign’ or ‘chemical’ drugs.^65^, ^66^, ^67^, ^68^ By framing their products as simply ‘unlocking the body's innate repair mechanisms’, grifters sidestep concerns about toxicity, regulation or mechanistic plausibility and exploit a deep cultural preference for the *natural* over the *synthetic*. This bias not only lowers patients' scepticism towards unproven biologics but also makes subsequent warnings from regulators or physicians seem counterintuitive—what could be risky about *your own* platelets or a ‘vitamin infusion’?

### Erosion of trust and structural access barriers

Declining trust in the medical establishment has created fertile ground for grifting.^69^, ^70^ Many patients—especially those with chronic or incurable illness—experience conventional care as inaccessible, impersonal or overly constrained by insurance, regulation and bureaucracy. Systemic inequities, long wait times and prior negative experiences can push individuals towards alternatives that promise attention, control and hope. In this context, grifters step in as apparent alternatives to a system perceived as uncaring or ineffective. Their messages resonate emotionally, especially in online groups that may reinforce scepticism towards medical institutions.

### Sociopolitical climate: Deregulation and institutional mistrust

The persistence of health‐related grift reflects more than individual bad actors—it is embedded in a broader sociopolitical climate. Deregulatory movements, scientific defunding and politicized scepticism of public institutions, healthcare professionals and biomedical journals have weakened oversight while emboldening grifters.^71^, ^72^, ^73^, ^74^ Pseudoscientific claims now flourish in an asymmetrical landscape where regulations are cast as barriers to ‘freedom’ and critiques are reframed as censorship. This allows pseudoscientific claims to persist, unchallenged by the usual rigors of scientific validation. Weakening regulatory authority in favour of ideology or consumerist deregulation invites exploitation and undermines decades of scientific progress in ensuring safety, efficacy and equitable access to legitimate medical innovation.

## THE ROLE OF UNFALSIFIABILITY

As philosopher Karl Popper posited, falsifiability is fundamental to distinguishing scientific claims from pseudoscience.^75^ For a claim to be scientific, it must be testable—and capable of being proven wrong. By contrast, pseudoscientific or illegitimate claims are often unfalsifiable: They lack clear hypotheses or measurable outcomes that could be disproven through experimentation.

Grifters often leverage unfalsifiability to insulate their products from scrutiny, claiming that their therapies provide benefits which would be difficult, or impossible, to challenge. Consider the assertion: ‘Our TPE protocol restores youthful plasma proteome balance and rejuvenates biological age, thereby extending lifespan’.^76^ These are vague concepts that cannot be, at least easily, scientifically evaluated. To challenge this claim, one would require a large, randomized, longitudinal trial lasting decades—an impractical, if not impossible, undertaking. In lieu of data, clinics offer testimonials and vague assurances of benefit, effectively shifting the burden of proof onto sceptics. By avoiding concrete, time‐bound outcomes and instead promising diffuse or future‐oriented benefits (e.g. ‘you'll feel younger’ or ‘you're investing in future health’), these actors sidestep accountability while maintaining the appearance of scientific legitimacy.

The persistence of such grift is not incidental—it thrives precisely because its claims cannot be conclusively refuted. Debunking misinformation demands rigorous studies, public engagement and institutional resources, while promoting pseudoscience may require nothing more than a viral video or targeted advertisement. Even when high‐quality evidence emerges to counter these claims, critics often dismiss it as biased, industry‐sponsored or irrelevant to their specific product.

### Responding to unfalsifiable claims

Healthcare providers, scientists and institutions can adopt strategies to counter unproven, unfalsifiable assertions.^77^ These may include:

- Acknowledge patient perspective: By listening to the patient's lived experience or understanding, the clinician and patient can connect, allowing for honest conversation.
- Reasserting the burden of proof: Emphasize that proponents must supply evidence. The absence of data (e.g. TPE for longevity) does not constitute evidence of efficacy.
- Highlight evidentiary gaps: Challenge the lack of evidence and highlight that no prospective trials or peer‐reviewed data exist and discuss the limitations of publications such as small case series or anecdotal reports
- Expose flawed logic: Claims such as ‘TPE improves biomarker X, therefore it extends lifespan’ represent a flawed causal inference—extrapolating from a surrogate end‐point without outcome data. This reasoning assumes, rather than demonstrates, clinical benefit.
- Seek specificity: Ask clarifying, empirical questions—‘How many years longer do patients live? Compared to what? Was there a control group?’
- Emphasize harm: Underscore the risks of delaying evidence‐based care, exposure to unsafe procedures and the erosion of regulatory safeguards.

## LEGITIMATE INNOVATION VERSUS GRIFTING

Distinguishing legitimate medical innovation from exploitative grift is essential. Not all novel therapies are suspect—many transformative advances in transfusion medicine, apheresis and cellular therapy began as bold ideas tested through careful, methodical inquiry. What separates innovation from grift today is not novelty, but process: transparency, oversight and scientific rigor.

Evidence‐based innovation follows a disciplined path. It begins with preclinical studies, undergoes ethical review, proceeds through phased clinical trials and is subjected to peer review, regulatory scrutiny and long‐term safety monitoring. Its development is cautious, accountable and based on reproducible evidence.

Grift, by contrast, bypasses these safeguards. It offers interventions marketed as cutting edge or ‘personalized’ without rigorous testing or validation. These offerings often lack institutional review, prospective data or safety oversight—and instead rely on branding, testimonials and selective interpretations of basic science.

While norms of medical practice allow for clinical discretion in off‐label use of reasonable therapies while awaiting conclusive evidence, this should be done to optimize patient outcomes rather than financial gain. To that end, professional medical societies and regulatory bodies must continue to offer guidance and support to treating providers to ensure best outcomes and guide public understanding.

Genuine breakthroughs—such as chimeric antigen receptor T‐cell therapy (CAR‐T), haematopoietic stem cell transplantation, gene therapies and pathogen‐reduction technology—represent true progress because they are supported by robust data and governed by ethical and regulatory frameworks. Professional medical societies should support legitimate work with mounting evidence and should receive funding support and guidance statements for appropriate use. Conflating these therapies with unproven stem cell injections or anti‐ageing plasma exchange not only misleads the public but also risks delegitimizing entire fields of impactful innovation.

## SYSTEMIC PRESSURES AND MORAL AMBIGUITY

It is understandable—perhaps even inevitable—that some patients seek care outside the boundaries of evidence‐based medicine, particularly when faced with chronic illness, limited treatment options or dissatisfaction with conventional care. Clinicians, too, are subject to systemic pressures that may blur professional judgement. Defensive medicine already drives overuse of questionable diagnostics and therapies.^78^, ^79^ As healthcare institutions confront financial instability, the appeal of cash‐based, biological ‘wellness’ services may intensify, especially when framed as personalized, cutting‐edge interventions.

In this context, some providers who were initially sceptical may grow more accepting over time, influenced by repetition, patient demand, discontent with mainstream medicine or by witnessing the revenue such services generate. The result is a morally ambiguous landscape—shaped by patient desperation, economic incentives and a shifting boundary between genuine innovation and commodified opportunism. Without clear policies, institutional safeguards and professional guidance, even well‐intentioned clinicians may find themselves complicit in practices that undermine scientific integrity and patient trust.

## REGULATORY FRAMEWORKS AND ENFORCEMENT

In the United States, human cells, tissues and cellular and tissue‐based products (HCT/Ps) fall under a two‐tier regulatory scheme. Products that meet all 21 CFR 1271.10(a) criteria (e.g. minimally manipulated, homologous use) are regulated solely under section 361, which requires establishment registration/listing, donor eligibility (screening and testing), Current Good Tissue Practice, labelling and adverse reaction and HCT/P deviation reporting to FDA.^80^, ^81^ Products that are more than minimally manipulated or used for nonhomologous indications are drugs/biologics that require premarket review under section 351.^80^, ^81^ FDA guidance delineates these boundaries; the agency's enforcement discretion for certain HCT/Ps ended on 31 May 2021, and subsequent appellate decisions have largely affirmed FDA authority.^82^

Enforcement remains uneven as some states create carve‐outs, complicating federal oversight and enabling cross‐border marketing.^83^ Beyond the FDA, the Federal Trade Commission has increased actions against deceptive advertising of regenerative therapies, including a 2025 ban and multimillion‐dollar penalties against the Stem Cell Institute of America and related entities.^84^ Professional societies provide practical guardrails; for example, the International Society for Stem Cell Research (ISSCR) guidelines oppose premature commercialization and outline criteria for ethical clinical translation; the International Society for Cell & Gene Therapy's (ISCT) Regulatory Affairs Committee details patient‐reporting pathways and cautions for exosomes/extracellular vesicles marketed as therapies; and the Association for the Advancement of Blood and Biotherapies (AABB) and the Foundation for the Accreditation of Cellular Therapy (FACT) and the Joint Accreditation Committee of the International Society for Cellular Therapy and the European Society for Blood and Marrow Transplantation (JACIE) standards offer accreditation frameworks for legitimate cellular therapy programmes.^85^, ^86^, ^87^, ^88^

## RECOMMENDATIONS FOR PRACTICE AND POLICY

Confronting the rise of biologics‐related grift requires more than individual vigilance—it demands coordinated institutional, regulatory and professional action. To ensure patient safety, preserve public trust and ensure scientific integrity in transfusion medicine, apheresis and cellular therapy, we propose the following multi‐tiered approach:

1. Institutional Safeguards
  - Develop formal policies that explicitly prohibit the use of unproven biological therapies outside research protocols.
  - Tie clinician remuneration and institutional support to compliance with evidence‐based standards.
  - Implement clinical review boards or ethics committees to evaluate and decline requests for interventions lacking scientific validity, ensuring consistent decision‐making.
2. Regulatory Reinforcement
  - Support policies that mandate clinical trials and peer‐reviewed publication for biological products and procedures labelled as medical and/or longevity treatments.
  - Advocate against deregulation efforts that equate consumer choice with unregulated access, emphasizing that protection from harm is a public good.
3. Patient and Provider Education
  - Institute standardized educational modules for clinicians on recognizing and responding to grift—covering marketing tactics, cognitive biases and managing difficult consultations.
  - Create patient‐facing materials—fact sheets, decision aids and media campaigns—that explain the difference between evidence‐backed therapies and unvalidated treatments, especially in high‐profile areas such as stem cell therapies or anti‐ageing plasma applications.
4. Transparent Oversight and Accountability
  - Encourage the creation of registry‐based post‐market surveillance for cellular therapies and other biologics used outside of clinical trials.
  - Partner with professional bodies to publish annual ‘rogue clinic’ reports (i.e. a list of facilities/clinics/sites that are providing unproven products/interventions), updating the public and providers about prevalent grifts and their associated harms.
5. Research and Funding Alignment
  - Allocate grant funding to studies that empirically evaluate unverified biological interventions, particularly those already widely marketed.
  - Offer infrastructure support to investigator‐initiated trials assessing common off‐label apheresis and stem cell therapies, enabling clarity on efficacy, harms and patient outcomes.
  - Sustained investment in rigorous, peer‐reviewed research and clinical trials (e.g. Canada's recent $33 million initiative by the Stem Cell Network to advance evidence‐based regenerative medicine across 36 projects and 14 disease areas) could help counter the growing marketplace of unproven biologics.^89^

## CONCLUSION

The proliferation of unproven medical products and interventions in transfusion medicine, apheresis and cellular therapy threatens both individual patient safety and the credibility of these specialties. These practices leverage sophisticated marketing, exploit cognitive biases and operate in an environment of eroding trust and regulatory uncertainty. Confronting these challenges requires healthcare professionals and institutions to reaffirm evidence‐based standards, promote scientific literacy and cultivate robust policies that protect patients. By delineating legitimate innovation from exploitation and actively countering unfalsifiable claims, we can preserve the integrity of medical science and ensure that advances in transfusion medicine and biotherapies serve the public good rather than commercial opportunism. It is imperative that institutions support medical professionals by developing clear policies, establishing escalation pathways and offering protection from retaliation when unproven requests are refused. This also demands the defence of strong, independent regulatory bodies. Without such stewardship, the line between innovation and exploitation collapses, placing patients and progress alike at risk. In the current moment, it is imperative that grifting be called out to prevent delegitimizing our fields and harming our patients.

## AUTHOR CONTRIBUTIONS

BDA: conceptualization, writing, supervision. SR: conceptualization, writing. VC: writing. ESA: writing. LDS: writing. GSB: conceptualization, writing, supervision. JWJ: conceptualization, writing, supervision.

## FUNDING INFORMATION

None.

## CONFLICT OF INTEREST STATEMENT

None.

## Data Availability

Data sharing not applicable to this article as no datasets were generated or analysed during the current study.
